# Bone metastasis in mammary cancer. A report of 10 cases in the female dog and some comparison with human cases.

**DOI:** 10.1038/bjc.1966.61

**Published:** 1966-09

**Authors:** W. Misdorp, B. A. den Herder

## Abstract

**Images:**


### BONE METASTASIS IN MAMMARY CANCER

### A Report of 10 Cases in the Female Dog and Some Comparison with Human Cases

W. MISDORP and B. A. DEN HERDER

*From the Department of Pathology, Netherlands Cancer Institute, Amsterdam, and the Department of Roentgenology, Free University, Amsterdam, Netherlands*

Received for publication April 25, 1966

MAMMARY cancer in the woman has been the subject of intensive study throughout a long period of years and the frequency of metastatic spread to the bones probably equals if not exceeds the frequency of metastasis to lungs and liver (Haagensen, 1956). There is general agreement that mammary cancer is the lesion most often responsible for osseous metastasis in the woman (Sutherland, Decker and Cilley, 1932 ; Lodwick, 1964).

The dog is the only other species in which bone involvement secondary to mammary cancer has been reported, and here published reports are few.

It is the purpose of this paper to present a new series of cases of malignant mammary neoplasia in the bitch in which metastasis to bone was demonstrated and to make some preliminary comparisons between the situation in the dog and in the woman.

### REVIEW OF LITERATURE

In the woman clinical signs connected with metastasis of mammary cancer are most frequently related to skeletal involvement (Gerbrandy *et al.*, 1959). Pain, especially in the back and extremities, often precedes radiological evidence of the metastatic lesion and is the most important clinical symptom associated with metastasis to bone (Copeland, 1931). Fractures of long bones, anaemia and hypercalcaemia occur, the latter condition often resulting in renal impairment and death (Sutherland *et al.*, 1932 ; den Ottolander *et al.*, 1957). Skeletal metastases recognized roentgenologically in women with advanced mammary cancer are mainly of an osteolytic type ; Sutherland (1932) reported 375 cases from a total of 393 and den Herder, Bosboom and Gerbrandy (1959) 122 amongst 160 cases. Detailed attempts to correlate roentgenological and autopsy findings made by Bachman and Sproul (1955) indicate, however, that roentgenograms do not give accurate information as the intertrabecular types of bone metastasis are missed.

About 180 cases of metastasized mammary tumours in dogs have been recorded in the literature (Misdorp, 1964) but little attention has been paid to the modes of dissemination. Dobberstein and Matthias (1942) and Braemer (1952) stated that no bone metastases had ever been recorded in cases of mammary cancer in dogs. Since then 9 cases of bone involvement due to metastatic mammary tumours (all carcinomas) have been reported in the literature. The data concerning these cases are listed in Table I.

TABLE I.—*Published Cases of Bone Metastasis Secondary to Mammary Cancer in the Dog*

| Author | Date | Number of dogs with metastasizing mammary cancer | Number of dogs with bone involvement | Type of tumour | Clinical signs | X-ray | Bone site | Metastasis elsewhere |
|---|---|---|---|---|---|---|---|---|
| Krook | 1954 | 77 | 4 | Carcinoma | — | — | ? | ? |
| Cotchin | 1954 | 21 | 2 | Carcinoma | — | — | Humerus femur | ? |
| von Sandersleben | 1958 | 21 | 1 | Adenocarcinoma | Moribund | — | Multiple | Lungs, liver |
| Nims *et al.* | 1961 | 1 | 1 | Adenocarcinoma | Paresis hind-quarters | Rarefaction + osteogenetic activity | Sixth lumbar vertebra | Lymphnodes, lungs, spleen, heart. |
| Owen | 1962 | 1 | 1 | Adenocarcinoma | Lameness | Provisional diagnosis osteosarcoma | Left humerus | Nowhere. |

The case described by von Sandersleben (1958) was a very remarkable one as 6 vertebrae as well as the skull, the pelvis and the four extremities proved to be involved. The dog described by Nims, Dean and Geil (1961) showed a tumour mass in the sixth lumbar vertebra roentgenologically. This tumour invaded the vertebral canal and grew around the trunks of the spinal nerves. Apparently this lesion had caused the paresis of the hindquarters observed clinically.

### REPORT

### *Ten Cases of Mammary Cancer in the Bitch with Metastasis to Bone*

Between January, 1960 and September, 1965 114 bitches with 120 metastasized mammary cancers were examined, clinically and pathologically. Eighty-eight disseminated carcinomas, 19 sarcomas and 13 carcino-sarcomas were found. In 10 of these animals there was involvement of the bone secondary to mammary cancer (Table II). In 6 cases the skeletal lesions had given rise to clinical symptoms, lameness being the main sign. The relationship between the presence of mammary tumours and symptoms of bone involvement was suspected clinically, however, in only 2 cases. In 3 bitches the metastatic lesions were detected

TABLE II.—*Ten Cases of Bone Metastasis Due to*

| File number (Breed) (Age) | Type of mammary tumour (Grade of malignancy*) | Clinical signs | Site of involved bones |
|---|---|---|---|
| 31488 . . . Breed unknown 12 years | Adenocarcinoma (arising in mixed tumour) Grade I | Increasing lameness R. hind leg, femoro-tibial joint enlarged | R. femur |
| 32390 . . . Crossbred 14 years | Adenocarcinoma Grade II | Emaciation, enlarged regional lymph node | 2nd lumbar vertebra (Fig. 5) |
| 32488 . . . Alsation 11 years | Anaplastic carcinoma Grade III | Increasing lameness R. hind leg 3 months after operation for mammary cancer | R. femur |
| 32571 . . . Unknown 13 years | Solid carcinoma Grade II | Large quickly growing tumour in the hip region | 2nd left rib (tumour in hip region adherent to periosteum of the femur) |
| 33342 . . . Crossbred 14 years | Adenocarcinoma Grade II | Pain in the back | 1st lumbar vertebra |
| 33561 . . . Spaniel Unknown age | Atypical squamous cell carcinoma of major teat duct Grade II | Lameness (some improvement after cortison-treatment) ; polyarthritis? nystachmus, opisthotonus | Ribs : multiple, metastases ; carpal and tarsal region ; mainly periostially and intra-articularly situated tumours |
| 63–169 . . Whippet 15 years | Solid carcinoma Grade II | Increasing lameness hind legs | Pelvis (os ischium). |
| 63–274 . . Crossbred Unknown age | Solid carcinoma Grade II | Lameness in fore and hind legs | Scapula, femur, pelvis (os ilium), 2nd right rib |
| 32521 . . . Breed unknown 7 years | Carcino-sarcoma | Severe dyspnoea, one year after operation for mammary cancer | 9th thoracic vertebra |
| 31489 . . . French Bulldog 13 years | Myxo-osteochondrosarcoma | Diabetes, emaciation, abdominal distension, melaena | 2nd lumbar vertebra |

*Grade of malignancy when " human " criteria (Scarff and Handley, 1938) were applied.

only when the spinal column was bisected longitudinally (this procedure was performed in nearly all the bitches with metastasized mammary cancer). In 5 dogs two metastasized mammary cancers were found. In three of these dogs metastasizing mammary osteosarcomas and mammary carcinomas (one adenocarcinoma, two solid carcinomas) were present. In two other dogs two carcinomas of different type had caused metastases.

In 5 bitches the local bone lesions were examined radiologically ; the other parts of the skeleton were not examined in this way (Table II). In 3 cases the provisional diagnosis of primary osteosarcoma was made (Fig. 1). In 2 other dogs the lesions were of the osteolytic type usually seen in the woman (compare Fig. 2 of a human case with Fig. 3 and 4 of a dog).

The bone lesions of the carcinoma group of 8 dogs (Table II), when examined histologically, proved to be predominantly of osteolytic type. These lesions were caused by several types of carcinoma mostly of solid or adenomatous type. Most of the carcinomas proved to be of the intermediate grade of malignancy (grade II) when " human " criteria of histological grading, used by Scarff and Handley (1938), were applied.

Three cases of our series which were rather unusual will be dealt with more extensively below. In one of these bitches the metastatic bone lesion was caused

*Mammary Cancer in the Bitch* (*Own Study*)

| X-ray diagnosis (a.m. = ante mortem examination p.m. = post mortem examination) | Microscopic type of bone metastases | Metastases elsewhere |
|---|---|---|
| m. + p.m. suspect for osteosarcoma (Fig. 1) | Mainly osteolytic type + extensive new bone formation | Adrenal only. |
| — | Osteolytic type | Regional lymph nodes, lungs (nodular type), spleen, liver, kidneys. |
| a.m. + p.m. suspect for osteosarcoma | Mainly osteolytic type + extensive periosteal new bone formation | Regional lymph nodes only. |
| — | Osteolytic type | Regional lymph nodes, lungs (nodular) and ovary. |
| — | Intertrabecular type | Regional lymph nodes, lungs (nodular + lymphangitic type) infiltration of the sacral nerve plexus. |
| p.m. destruction of ribs ; shafts of the long bones intact | Ribs : osteolytic type | Regional lymph nodes, lungs (nodular type), spleen, cerebellum. |
| p.m. suspect for osteosarcoma | Osteolytic type + extensive periosteal new bone formation | Regional lymph nodes, lungs (lymphangitic type) |
| p.m. osteolytic lesions suggestive for metastases (Fig. 3 and 4) | Osteolytic type | Regional lymph nodes, lungs (nodular type), liver. |
| — | Mainly osteolytic type (adenocarcinoma + chondrosarcoma) | Lungs (nodular type), kidneys, heart, spleen. |
| — | Intertrabecular type (Fig. 6) | Regional lymph nodes, lungs (nodular type), pleura, spleen, mesentery, kidneys, pancreas. |

by a mammary sarcoma, in another bitch by a carcino-sarcoma. These cases are unique because in the literature no reports concerning bone metastasis due to these types of mammary tumours in the dog or in the woman have been found. In the third dog the type of tumour, an atypical squamous cell carcinoma, and the way of spread seem to be uncommon.

*Dog* 31489, *French Bulldog bitch, aged* 13 *years*

This animal was euthanized after a long history of diabetes mellitus, emaciation, melaena and distention of the abdomen with palpable firm tumours. One year before euthanasia a chain of quickly growing ulcerating bony mammary tumours had been removed. In the right axillary and right superficial inguinal lymph nodes, as well as in the sternal and para-aortic lymph nodes, large bony tumours could easily be detected. In the lungs and pleura and also in the kidneys, liver, mesentery and pancreas large bony tumours were present. The body of the second lumbar vertebra showed an ill-defined grey-white area on longitudinal section.

*Microscopically* the tumours in the right axillary lymph node and in the lungs showed the picture of rather highly differentiated fibrous and myxoid tissue, cartilage and bone. The vascular emboli in the liver and the tumourous structures in the right inguinal lymph node showed a more cellular sarcoma-like pattern. In the lumbar vertebra the bone marrow was replaced by cartilage in which different degrees of maturation were recognized (intertrabecular type, Fig. 5). The appearance of the highly differentiated and widely disseminated mesenchymal tumour tissue in this dog closely resembled that previously observed in some cases of canine mammary sarcoma. It seems very likely that one or more of the previously removed, quickly growing tumours (not examined histologically) had caused the extensive tumour formation in the two regional lymph-nodes and in the distant organs.

*Dog* 32521, *breed unknown, bitch, aged* 7 *years*

One year before euthanasia the bitch had been operated on for a recurrent mixed mammary tumour consisting of papilliferous growing epithelium and myxomatous and chondroid tissue. The epithelium as well as the mesenchymal

---

EXPLANATION OF PLATES

FIG. 1.—Dog 31488 (X-ray picture). Metastasis in the right femur showing extensive periosteal new bone formation simulating primary osteosarcoma.

FIG. 2.—Woman (X-ray picture). Osteolytic metastasis in the pelvis from a mammary carcinoma (compare with Fig. 3 and 4).

FIG. 3.—Dog 63–274 (X-ray picture). Osteolytic metastases (arrows) in the humerus secondary to a mammary carcinoma.

FIG. 4.—Dog 63–274 (X-ray picture). Osteolytic metastases (arrows) in the scapula, secondary to a mammary carcinoma.

FIG. 5.—Dog 31489. Intertrabecular type of bone metastasis in the second lumbar vertebra due to a mammary sarcoma. Note the highly differentiated chondrosarcomatous tissue. Staining: haematoxylin-azophloxin. ×57.

FIG. 6.—Dog 32521. Carcino-sarcoma. Neoplastic epithelium in close contact with neoplastic osteoid and bone in the primary mammary tumour. Staining: haematoxylin-azophloxin. ×160.

FIG. 7.—Dog 33561. Atypical squamous cell carcinoma (" metaplastic carcinoma ") showing extensive cornification from the primary mammary tumour. Staining: haematoxylin-azophloxin. ×34.

1

2

Misdorp and den Herder.

3

4

5

6

7

tissue proved to be rather highly differentiated histologically. The dog was destroyed because of increasing dyspnoea, distension of the abdomen, anorexia and emaciation. At autopsy the lungs contained two large hard tumours, a smaller and softer one and several small bony lesions. The spleen contained one very large (22 × 16 × 16 cm.) partly soft and partly bony tumour and several smaller ones. The kidneys showed several round bony nodules and the heart contained one firm white nodule. The ninth thoracic vertebra showed an area of soft white tissue on longitudinal section.

*Microscopically* the tumours in the lung, the spleen, the kidney and the heart were partly of epithelial or mesenchymal type, partly of mixed type. The mesenchymal tissue especially was highly differentiated as in dog 31489. The tumour tissue in the ninth thoracic vertebra had destroyed many bony trabeculae (osteolytic type) and consisted of rather highly differentiated carcinomatous and sarcomatous tissue situated closely together (Fig. 6).

*Dog* 33561, *Spaniel, bitch, age unknown*

This bitch had been operated on for a mammary tumour in the fourth and fifth left inguinal glands (6 × 4 × 2½ cm.) which was partially situated in the teat canal.

*Microscopically* the tumour proved to be a solid carcinoma with extensive cornification and areas with ghost cells. The picture in some parts resembled that of Malherbe's tumour. Eight months after the operation the dog was destroyed because of increasing lameness, nystagmus, opisthotonus and difficult respiration. The lameness, which seemed to be due to arthritis of the swollen left carpal and tarsal joints, initially, had subsided when treated with cortisone.

*Post-mortem examination* revealed a large recurrent mammary tumour (Fig. 7), numerous large round metastatic nodules in the lung, destructive tumourous tissue in some ribs, tumours in and around the left carpal and tarsal joint and a relatively large tumour in the cerebellum. These tumours showed a histological picture similar to that of the operated mammary tumour. We made an arbitrary diagnosis of atypical squamous cell carcinoma of the teat canal.

### DISCUSSION

The volume of material studied in the dog is small when compared with the statistical data available concerning mammary cancer in the woman and preliminary conclusions only can be drawn. Bone involvement in 8 out of 10 dogs in our series was due to metastatic mammary carcinoma. The frequency of bone involvement in bitches with mammary carcinoma (8 out of 88) is distinctly lower than that recorded in the woman. However, it should be noted that often dogs with mammary cancers are euthanized before the terminal stage is reached. It might be that metastasis, e.g. to bone, in these dogs was still incomplete. Multiple bone lesions were found in only 2 dogs ; in the woman multiple lesions are common. In 2 dogs, apart from actual involvement of bone itself, metastatic deposits in the periosteal tissue were found without lesions in the underlying cortex. These findings are very uncommon in the woman. Three lesions in the bone were suspected to be primary osteosarcomas roentgenologically because of the extensive periosteal new bone formation together with the osteolytic foci in the shaft (Fig. 1). Two of these lesions were situated in a long bone (femur)

and resembled that of Owen (1962) and of Cotchin (1958) who reported on a bone metastasis in the femur secondary to a tonsillar carcinoma. In the woman " in both osteolytic and osteoblastic types of bone metastases, periosteal response when present is usually scant " (Lodwick, 1964).

Possibly the periostium of the long bones in dogs is more prone to proliferation. In this respect it should be noted that hypertrophic osteo-arthopathy (Pierre-Marie's disease) occurs rather frequently in dogs. In our series of dogs the mammary carcinomas had spread to parts of the so-called axial skeleton. The axial skeleton where red bone marrow persists, e.g. the spongy bones of the pelvis, vertebrae, rib and the proximal ends of the humerus and femur, forms also the site of predilection in the woman (Copeland, 1931 ; Sutherland, 1932 ; Fort, 1935).

In our dogs the carcinomas, which were of different types, had given rise to osteolytic lesions. In the cortex in the woman osteolytic metastases are often caused by the highly malignant undifferentiated mammary carcinomas, whereas the slowly growing carcinomas, e.g. adenocarcinomas, are often connected with the osteoblastic type (Sutherland, 1932 ; Meyer, 1957).

### SUMMARY

Ten cases of bone metastasis in mammary cancer in the bitch are reported. These 10 cases form part of a series of 114 bitches with 120 disseminated mammary cancers.

The literature concerning bone metastasis in mammary cancer in the dog and the woman is briefly reviewed.

The extent of bone involvement in the bitch seems considerably lower than that recorded in the woman, although in 7 of 10 cases reported here the so-called axial skeleton was found to be involved as in the woman. The bone metastases in these dogs simulated osteosarcoma roentgenologically and clinically, and in several cases extensive periosteal new bone formation was present.

The lesions, which were caused by several kinds of mammary carcinoma in 8 dogs, were mainly of osteolytic type.

Three cases are reported in greater detail. Two of these concern bone metastasis due to mammary sarcoma and carcino-sarcoma, respectively, and which have not apparently been previously reported in the medical or veterinary literature.

Our thanks are due to Professor Teunissen, Director of the Small Animal Clinic, Utrecht, and the colleagues, Gajentaan, Koopmans, Kraan and Pijnappel, for their co-operation. We also are indebted to Mrs. Hijtman, Hol and Korver for technical assistance and to Miss Van Gelder for typing the manuscript. We wish to thank Professor Cotchin and Dr. Appleby, Royal Veterinary College, London, for their assistance in producing this paper. We are also indebted to the Clinical Staff of the Netherlands Cancer Institute and especially Professor Hampe for his encouragement and criticism.

### REFERENCES

BACHMAN, H. L. AND SPROUL, E.—(1955) *Bull. N.Y. Acad. Med.*, **31**, 146.
BRAEMER, CH.—(1933) Thesis, Doct. Vet., Leipzig.
COPELAND, M. M.—(1931) *Radiology*, **16**, 198.

Cotchin, E.—(1958) *Vet. Rec.*, **70**, 752.—(1954) *Br. vet. J.*, **110**, 218.
Dobberstein, J. and Matthias, D.—(1942) *Arch. wiss. prakt. Tierheilk.*, **78**, 18.
Fort, W. A.—(1935) *Radiology*, **24**, 96.
Gerbrandy, J., Hampe, J. F., Lokkerbol, H. and van Slooten, E. A.—(1959) *Ned. Tijdschr. Geneesk.*, **103**, 559.
Haagensen, C. K.—(1956) 'Diseases of the breast'. Philadelphia and London (W. B. Saunders).
den Herder, B. A., Bosboom, B. J. M. and Gerbrandy, J.—(1959) *Jaarb. Kankeronderz. Ned.*, **9**, 57.
Krook, L.—(1954) *Acta path. microbiol. scand.*, **35**, 407.
Lodwick, G. S.—(1964) 'Tumors of Bone and Soft Tissue'. Chicago (Year Book Medical Publishers), pp. 243–268.
Meyer, P. C.—(1957) *Br. J. Cancer*, **11**, 509.
Misdorp, W.—(1964) Thesis, Doct. Vet., Utrecht.
Nims, J., Dean, E. E. and Geil, R. G. J.—(1961) *Am. J. vet. Med.*, **138**, 2.
den Ottolander, G. J. H., Hellendoorn, H. B. A., de Jager, H. and Gerbrandy, J.—(1957) *Ned. Tijdschr. Geneesk.*, **401**, 2066.
Owen, L. N.—(1962) *Vet. Rec.*, **74**, 439.
von Sandersleben, J.—(1958) *Mh. Tierheilk.*, **11**, 191.
Scarff, R. W. and Handley, R. S.—(1938) *Lancet*, ii, 582.
Sutherland, C. G., Decker, F. H. and Cilley, E. I. L.—(1932) *Am. J. Cancer*, **16**, 1457.


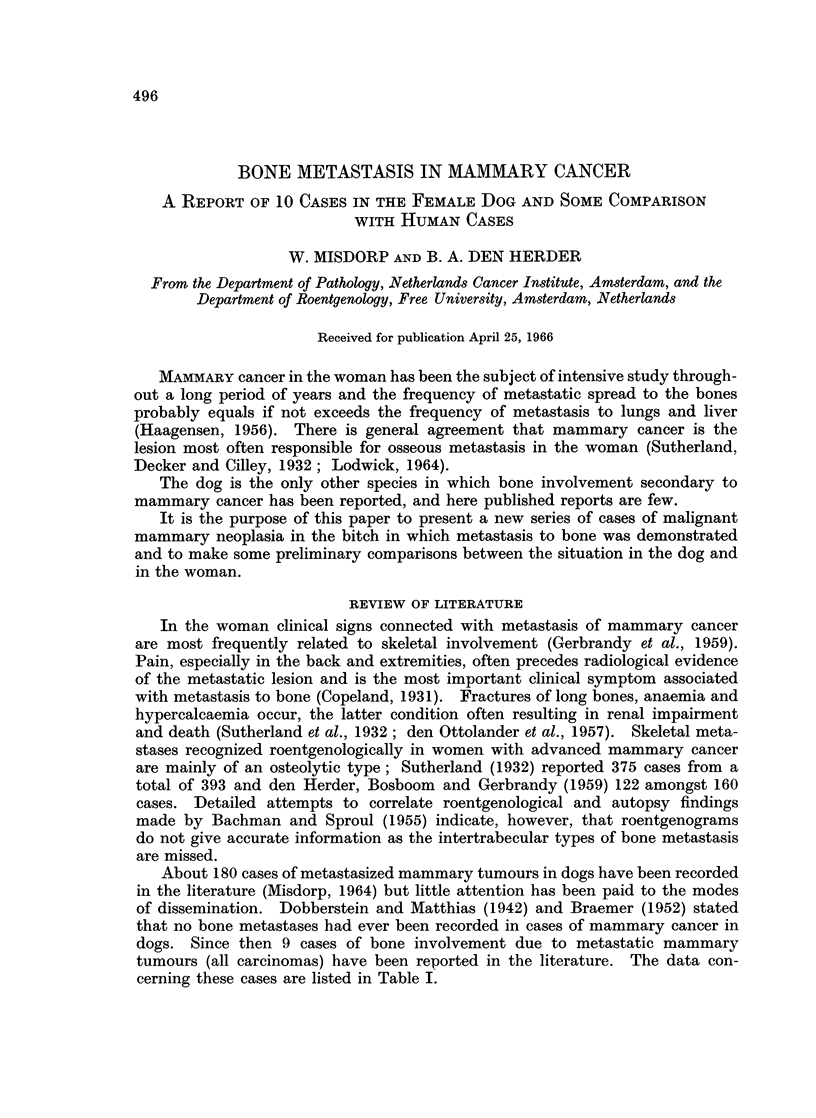

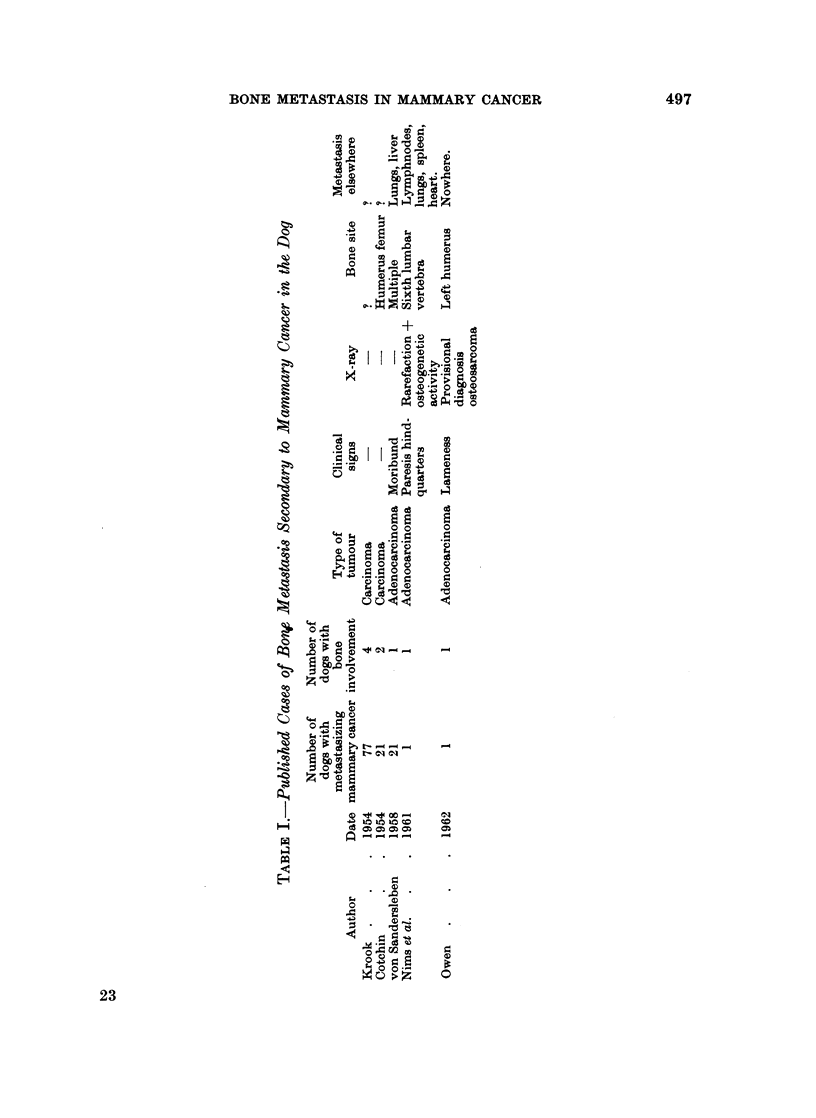

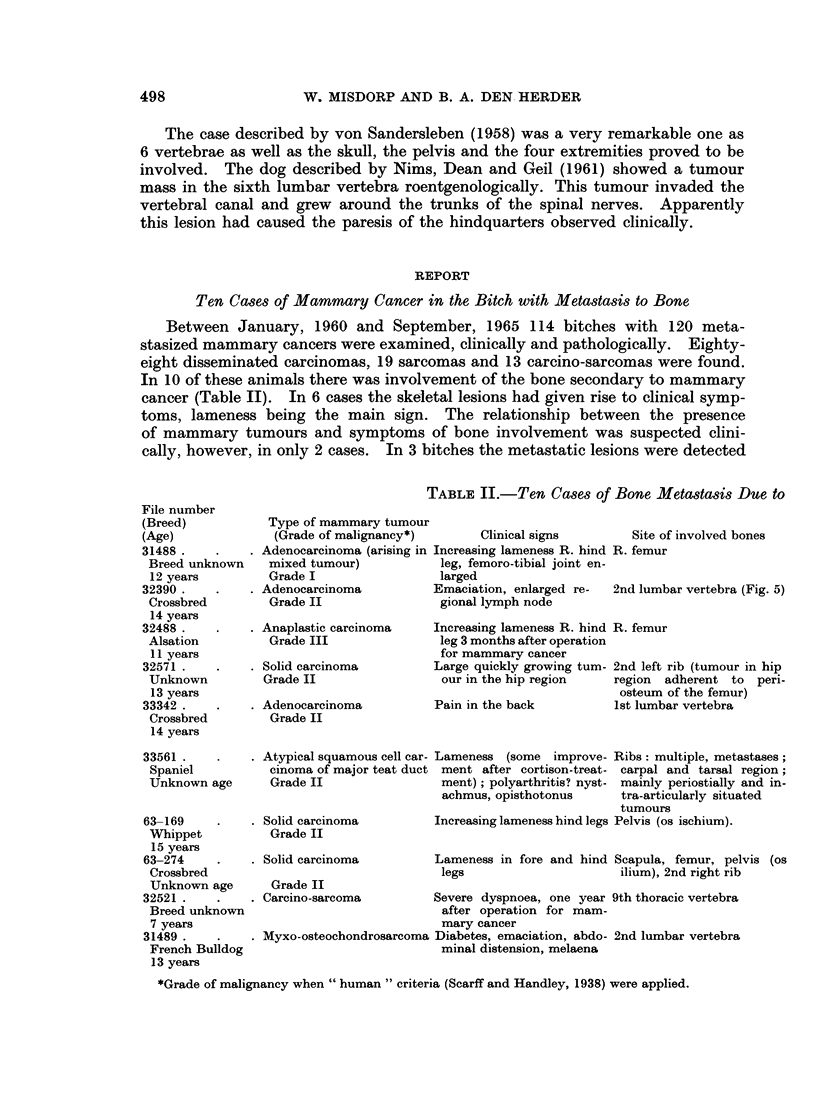

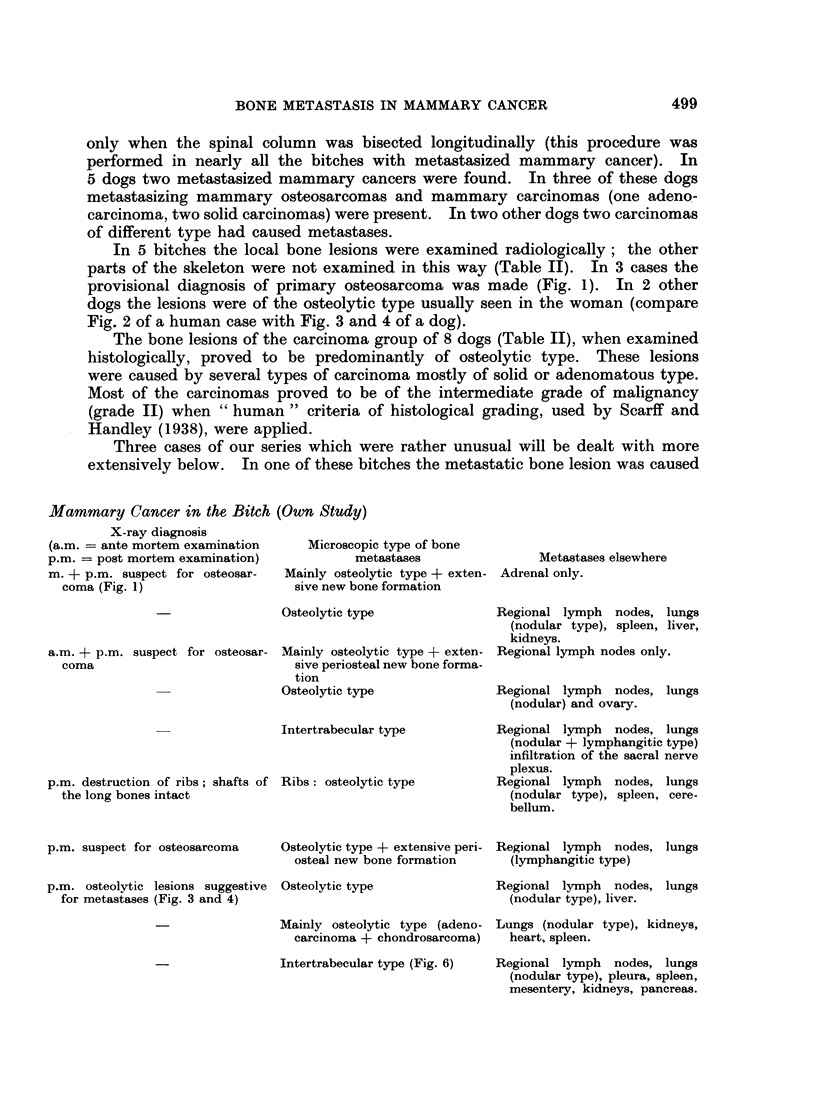

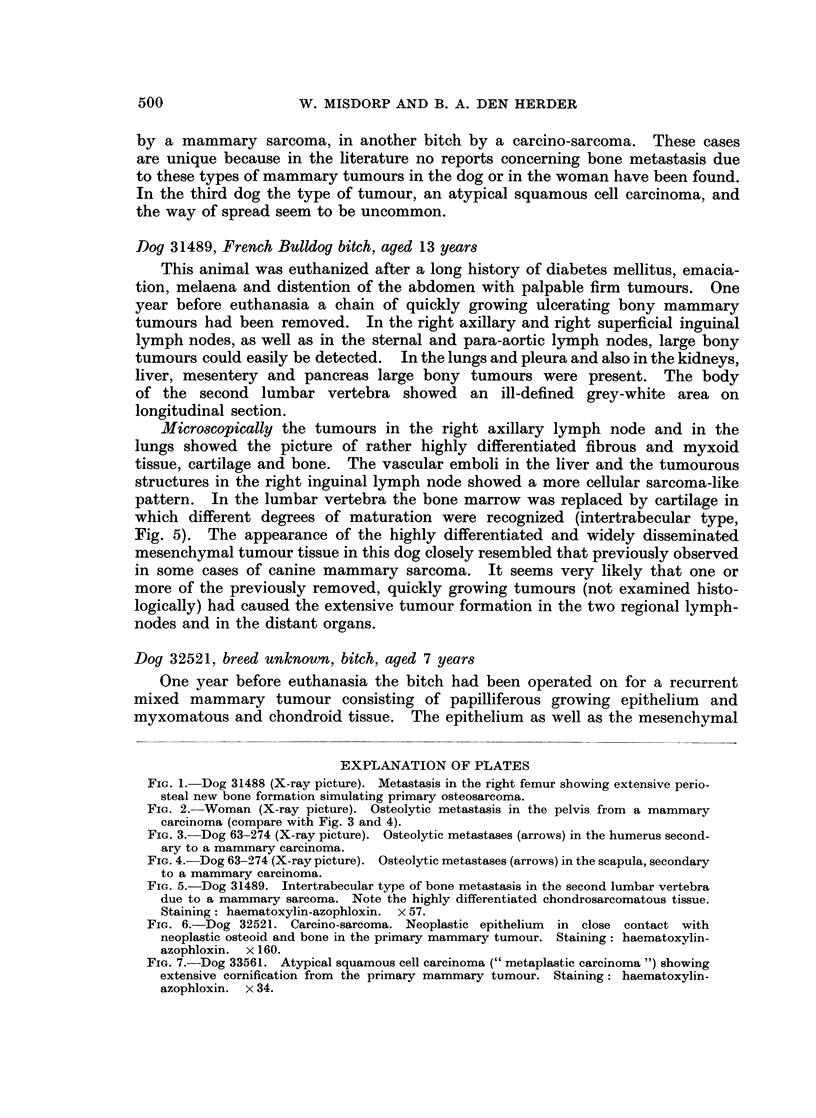

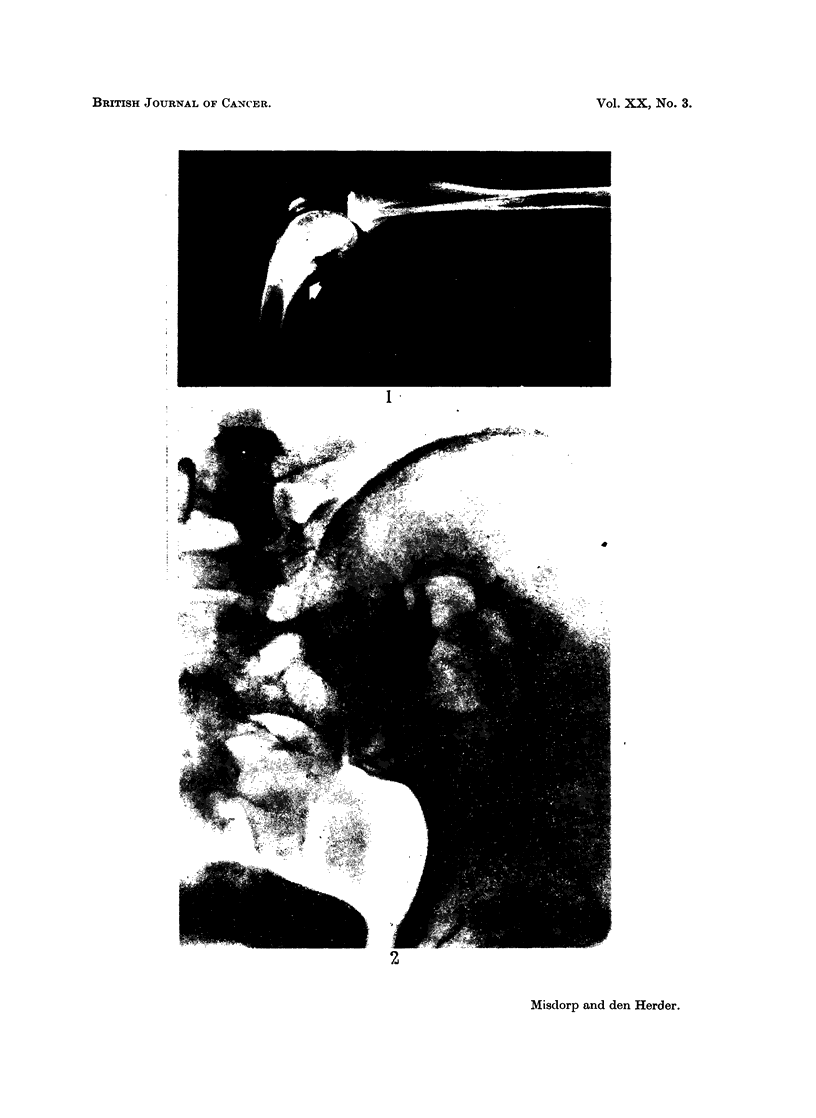

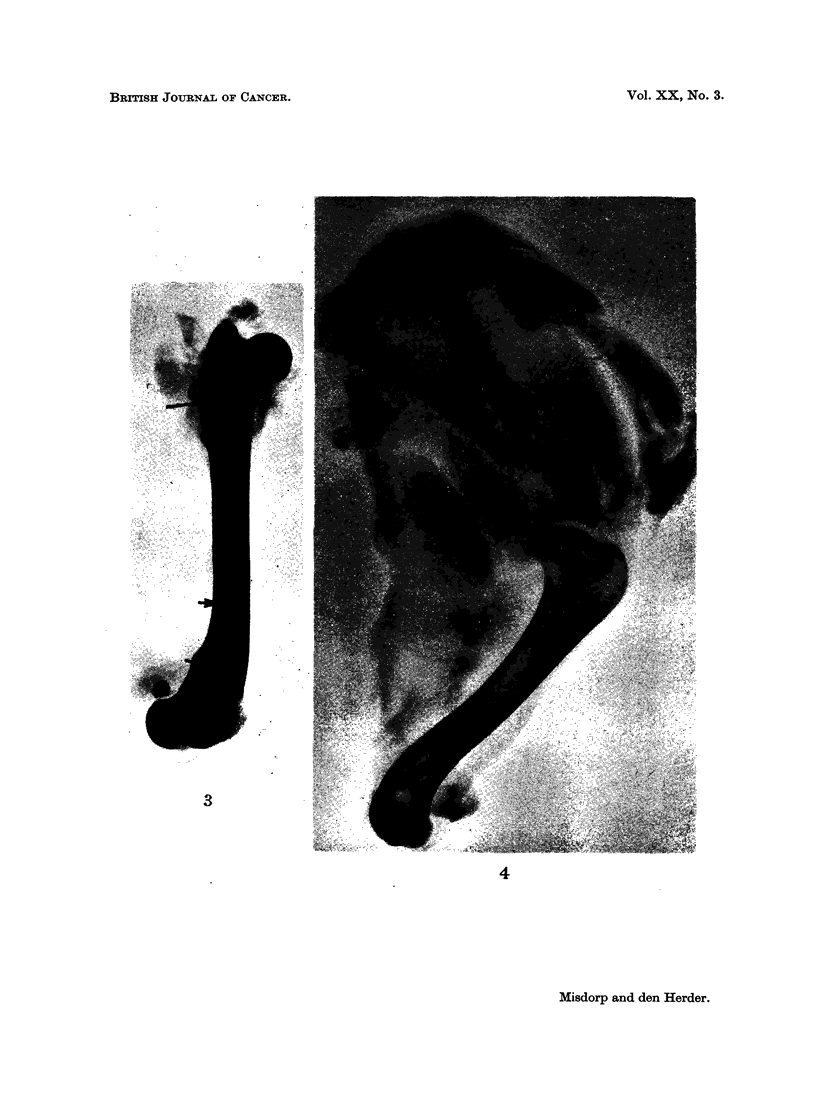

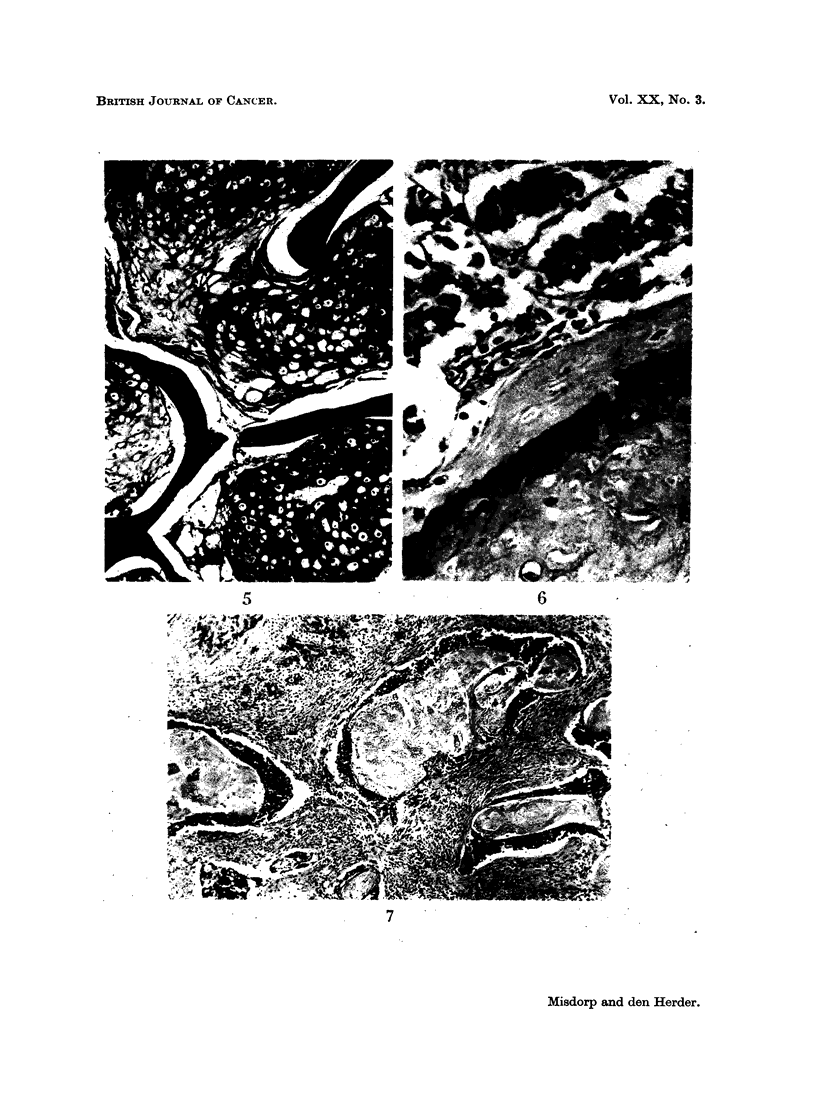

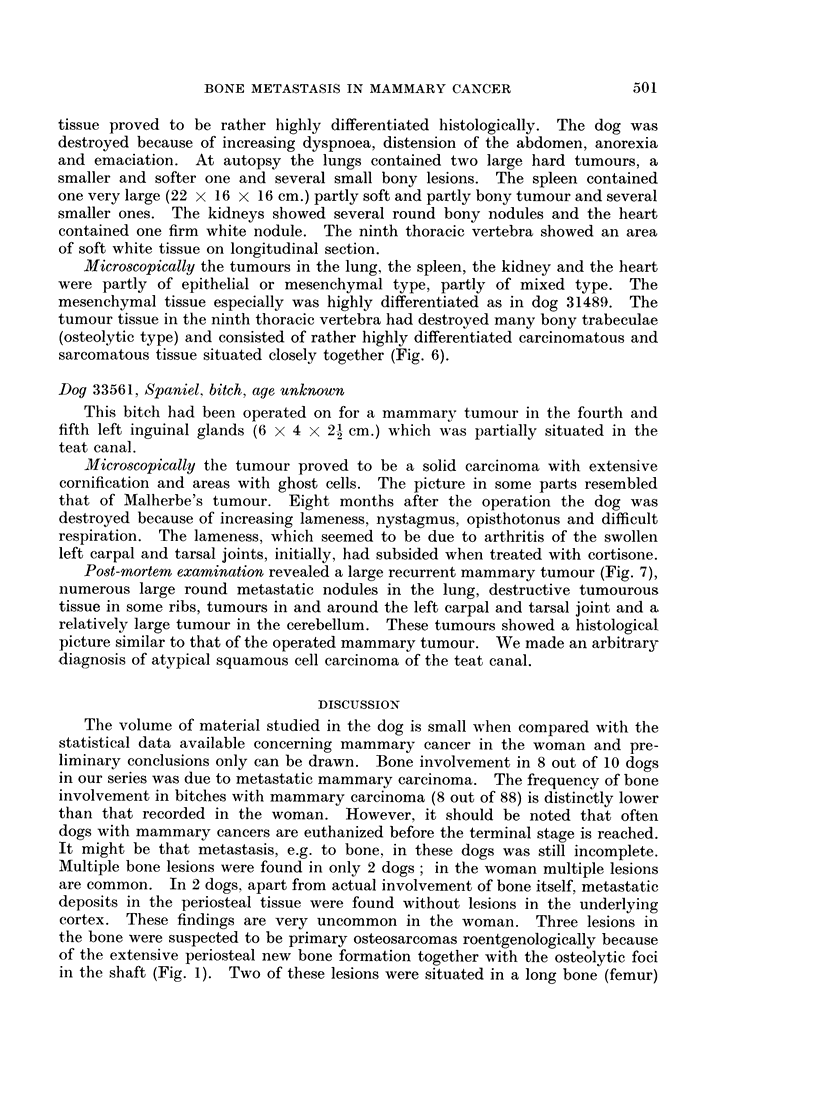

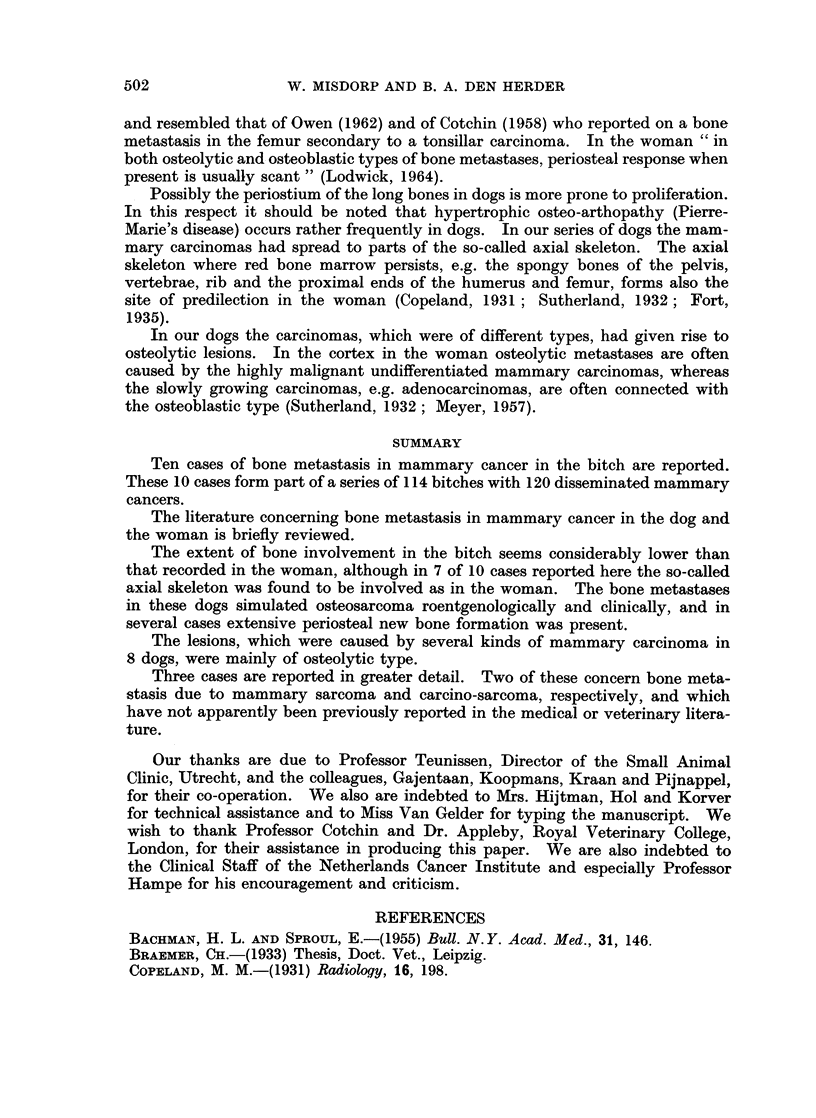

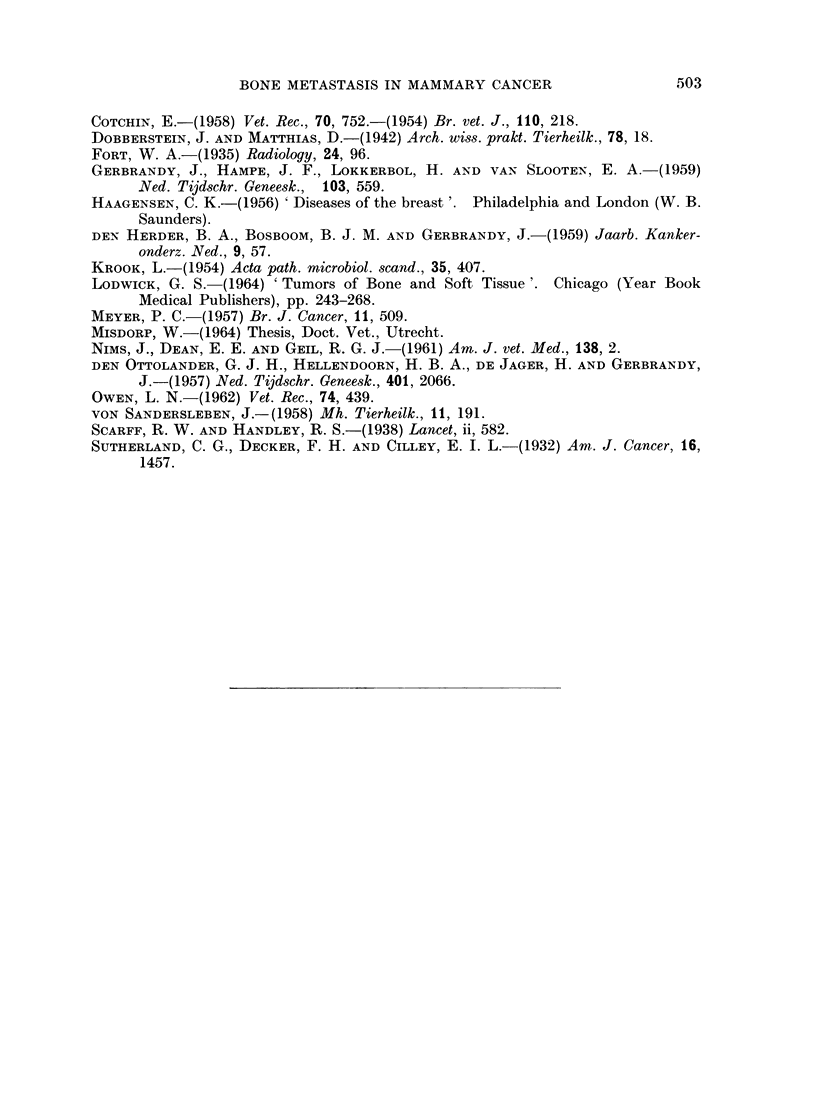


## References

[OCR_00607] DEN OTTOLANDER G. J., HELLENDOORN H. B., DE JAGER H., GERBRANDY J. (1957). Acute hypercalciëmie bij patiënten met osteolytische metastasen van mammacarcinoom.. Ned Tijdschr Geneeskd.

[OCR_00583] GERBRANDY J., HAMPE J. F., LOKKERBOL H., VAN SLOOTEN E. A. (1959). Klinische diagnostiek van de metastasering van gezwellen.. Ned Tijdschr Geneeskd.

[OCR_00597] KROOK L. (1954). A statistical investigation of carcinoma in the dog.. Acta Pathol Microbiol Scand.

